# A Critical Review of Impact of Ureteral Access Sheath Use on Renal Stone Clearance During Flexible Ureterorenoscopy: A Retrospective Cohort Study

**DOI:** 10.1002/hsr2.73013

**Published:** 2026-08-09

**Authors:** Asad Ali, Faizan Ahmed

**Affiliations:** ^1^ Department of Medicine Sheikh Khalifa Bin Zayed Al Nayhan Medical and Dental College Lahore Pakistan

Dear Editor,

We read with great interest the recent article “Impact of Ureteral Access Sheath Use on Renal Stone Clearance During Flexible Ureterorenoscopy: A Retrospective Cohort Study” by Al‐Zubi et al. [1] evaluating the impact of Ureteral Access Sheath (UAS) use on renal stone clearance during Flexible Uretero‐renoscopy (FURS). While the authors acknowledged several important limitations, we believe additional methodological concerns warrant consideration when interpreting the reported association between UAS use, shorter operative time, and higher stone‐free rates.

First, the study relied solely on univariate comparisons without performing multivariable regression analysis or propensity‐score adjustment. Although baseline characteristics such as stone size, density, and number appeared comparable between groups, several established determinants of FURS outcomes—including renal collecting system anatomy, infundibulopelvic angle, degree of hydronephrosis, ureteral caliber, body mass index, and surgeon experience—were not evaluated or adjusted for. Consequently, residual confounding may have influenced the observed superiority of UAS, making it difficult to determine whether the improved stone‐free rate was attributable to the sheath itself or to unmeasured patient‐ and procedure‐related factors. This limitation is addressed in the article by Traxer et al. [2]

Second, stone burden was characterized primarily by maximal stone diameter rather than volumetric assessment. Stone volume has been shown to be a more accurate predictor of operative complexity and postoperative stone‐free status than linear dimensions alone. Patients with similar maximum stone diameters may have substantially different total stone burdens, particularly in cases involving multiple calculi or irregularly shaped stones. Failure to account for stone volume may therefore have introduced bias into the comparison of operative time and stone clearance outcomes between the two groups. This limitation can be addressed by incorporating computed tomography‐based volumetric measurements to provide a more precise assessment of stone burden, as in the article by Xun et al. [3].

Third, although stone location was reported, no subgroup analysis according to stone position was performed. Nearly half of the included stones were located in the lower calyx, a location known to be associated with lower stone‐free rates because of unfavorable anatomical factors and restricted scope deflection. Unequal distribution of anatomically challenging stones between groups could have significantly influenced postoperative outcomes. This limitation can be addressed by stratified analyses according to stone location and renal anatomical characteristics that provide a more robust evaluation of the true impact of UAS on procedural success, as in the article by Berquet et al. [4].

Finally, the study did not report surgeon‐related variables or account for the learning curve associated with flexible ureteroscopy. Operator experience is a well‐recognized determinant of operative efficiency and stone‐free outcomes. If UAS was preferentially utilized by more experienced surgeons or during later phases of the learning curve, the observed reductions in operative time and improvements in stone clearance may partially reflect operator proficiency rather than the effect of the access sheath itself. The study should control for surgeon experience or employ standardized operative protocols to minimize this potential source of bias, as in article by Somani et al. [5]

Taken together, these factors may have contributed to the observed differences in operative time and stone‐free rates and should be considered when interpreting the conclusion that UAS independently improves outcomes during flexible ureterorenoscopy.

## Author Contributions

**Asad Ali:** conceptualization, writing – review and editing, writing – original draft, project administration. **Faizan Ahmed:** writing – original draft, writing – review and editing, data curation, visualization, conceptualization.

## Funding

The authors have nothing to report.

## Ethics Statement

The authors have nothing to report.

## Conflicts of Interest

The authors declare no conflicts of interest.

## Data Availability

Data sharing is not applicable to this article as no datasets were generated or analyzed during the current study.
